# Application of combined multiple organ resection in the treatment of locally advanced gastric cancer

**DOI:** 10.1097/MD.0000000000044020

**Published:** 2025-08-29

**Authors:** Beibei He, Shengwen Li, Yaming Guo, Song Li, Wenjun Zhu, Xinyi Wang, Baozhang Chen, Shile Wu

**Affiliations:** a Department of General Surgery, Qinghai Provincial People’s Hospital, Xining City, Qinghai Province, China.

**Keywords:** combined resection, locally advanced gastric cancer, multiple organs, prognosis, surgical indications

## Abstract

This study analyzes the application value of extended multi-organ resection in the treatment of locally advanced gastric cancer, and to provide reference for the clinical diagnosis and treatment of locally advanced gastric cancer patients. From January 2016 to April 2022, 64 cases of locally advanced gastric cancer admitted to our hospital were analyzed retrospectively. Combined multiple organ resection was used as the treatment strategy, and the general information and surgical data of patients were analyzed to evaluate the prognosis of extended multi-organ resection in the treatment of locally advanced gastric cancer and the factors affecting these clinical outcomes. A total of 64 patients with locally advanced gastric cancer were included, including 34 males and 30 females, with an average age of 57.28 ± 12.27 years. Among them, combined pancreatectomy was performed in 25 cases, splenectomy in 20 cases, hepatectomy in 12 cases, pancreaticoduodenectomy in 5 cases, pancreaticoduodenectomy and right colon resection in 1 case, and colon resection in 1 case. The average operation time was 159.64 ± 25.19 minutes and the median intraoperative bleeding was 150 mL. All 64 patients achieved R0 resection, and all patients were followed up. Postoperative complications occurred in 18 cases, of which the mortality rate was 16.67% (3/18). The 1-year, 3-year, and 5-year survival rates were 59.4%, 35.9%, and 14.6%, respectively. It is feasible to treat locally advanced gastric cancer by extended combined organ resection. As long as the indications are strictly grasped, the overall prognosis of patients can be improved.

## 1. Introduction

Gastric cancer is one of the most common cancers in the world. According to the global cancer data report published on *CA: A Cancer Journal for Clinicians* in 2021, gastric cancer ranks fifth in the global cancer incidence rate and fourth in the mortality rate, among which East Asia is the high incidence area of gastric cancer, and China accounts for about half of the cases in East Asian countries.^[1,2]^ At present, locally advanced gastric cancer still accounts for a considerable proportion in China, and most of them invade adjacent organs. Most patients have reached locally advanced stage when they are diagnosed.^[3]^ For this type of patients, the prognosis of palliative resection or nonsurgical treatment (such as radiotherapy and chemotherapy) is often extremely poor, and the postoperative recurrence rate and mortality rate have been at a high level.^[4,5]^ It is generally believed that R0 resection should still be regarded as the main goal in advanced gastric cancer, and extended multi-organ resection (EMR) should be adopted if necessary.^[6,7]^ However, at present, it is still controversial whether to perform extended combined organ resection for locally advanced gastric cancer invading adjacent organs and the prognosis and survival benefit after operation. In this study, the clinical data of patients with locally advanced gastric cancer who received EMR were retrospectively analyzed, and the safety and feasibility of EMR in the treatment of locally advanced gastric cancer were analyzed and discussed, so as to provide reference for the formulation of clinical surgical plans for such patients.

## 2. Materials and methods

### 2.1. Data collection

The general data of EMR patients admitted to our hospital from January 2016 to April 2022 were analyzed retrospectively. Inclusion criteria: Patients diagnosed with locally advanced gastric cancer and receiving at least 2 organ resections including gastrectomy; Clinical records and follow-up data are complete. Exclusion criteria: Patients who refused surgical treatment; The medical records are missing or lost. The diagnosis of cancer staging is based on clinical data, including CT, endoscopic ultrasonography and postoperative pathology. This study was conducted according to the ethical principles of medical research involving human subjects in Helsinki Declaration. Patients and their families signed informed consent before operation, and EMR was recommended for all patients with locally advanced gastric cancer who were suspected of invading adjacent organs.

### 2.2. Treatment method

**Figure 1.**
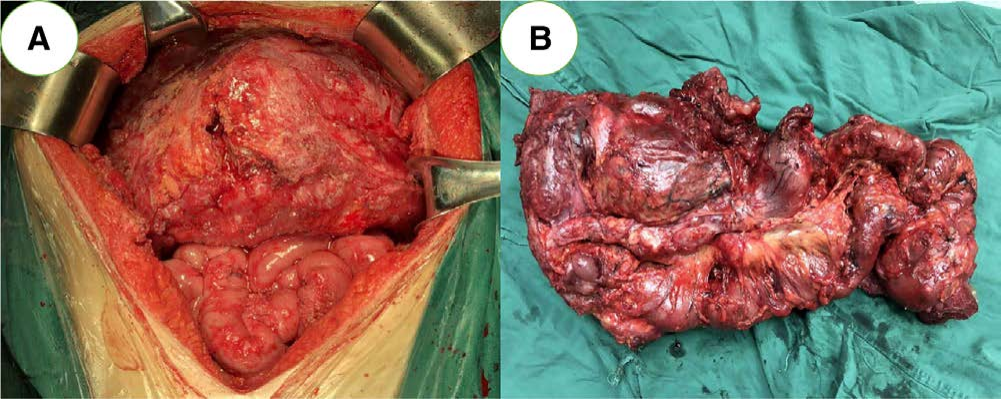
EMR intraoperative image. The patient suffered from colon obstruction due to gastric cancer invading the pancreas and transverse colon liver area. After multidisciplinary consultation before operation, he underwent resection of huge abdominal tumor + total gastrectomy + splenectomy + resection of pancreatic body and tail + right hemicolectomy + jejunostomy. EMR = extended multi-organ resection.

All surgical operations were performed by senior surgeons, and the adopted definition of EMR refers to the simultaneous resection of the whole stomach and the invaded organs. The standard surgical methods for locally advanced gastric cancer for the purpose of cure include D2 resection of gastric cancer and resection of affected organs.^[8]^ One representative case of advanced gastric cancer treated with EMR was diagnosed as gastric cancer invading the pancreas and transverse colon liver area and complicated with colon obstruction before operation. After multidisciplinary consultation before operation, the patient was treated with huge abdominal tumor resection + total gastrectomy + splenectomy + pancreatic tail resection + right hemicolectomy + jejunostomy, and was followed up regularly after operation. The patient is still alive at the fifth year after operation (as shown in Fig. 1).

### 2.3. Follow-up

All postoperative patients were followed up regularly every 3 to 6 months by outpatient service, SMS, telephone, and WeChat. The contents of follow-up include the current general situation of the patient, whether there are postoperative complications and the survival status of the patient. The deadline for follow-up is July 6, 2022, and the end point is the death of the patient or the deadline for follow-up.

### 2.4. Statistical analysis

SPSS 26.0 and GraphPad Prism 8.0 were used to analyze the data statistically. The measurement data of normal distribution is expressed by the mean standard deviation, and the measurement data of non-normal distribution is expressed by the median. Kaplan–Meier method is used to calculate the survival rate and draw the survival curve.

## 3. Results

### 3.1. General information

**Figure 2.**
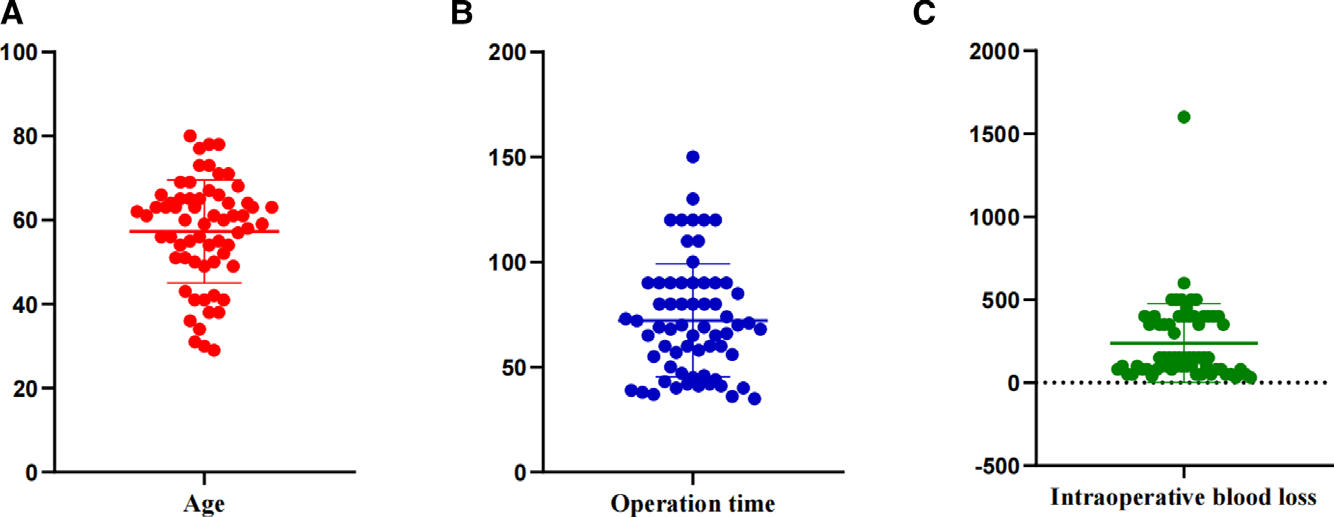
Distribution of key patient characteristics. (A) Age (yr); (B) Operation time (min); (C) Intraoperative blood loss (mL).

A total of 64 patients met the inclusion criteria, including 34 males and 30 females, with a median age of 60 years. Among them, 2 cases were complicated with intestinal obstruction, 26 cases with pyloric obstruction, 5 cases with gastrointestinal bleeding and 3 cases with gastrointestinal perforation. Combined pancreatectomy was performed in 25 cases, splenectomy in 20 cases, hepatectomy in 12 cases, pancreatoduodenectomy in 5 cases, pancreatoduodenectomy combined with right hemicolectomy in 1 case and colectomy in 1 case. The operation time was 120 to 266 minutes, with an average of 159.64 ± 25.19 minutes; The intraoperative blood loss was 30 to 1600 mL, with a median of 150 mL. Postoperative pathological results showed that there were 38 cases of adenocarcinoma (including 20 cases of poorly differentiated adenocarcinoma, 12 cases of moderately differentiated adenocarcinoma and 6 cases of highly differentiated adenocarcinoma), 13 cases of signet-ring cell carcinoma, 7 cases of adenosquamous carcinoma, 3 cases of squamous carcinoma and 3 cases of undifferentiated carcinoma. TNM staging results: The number of cases in stage II, III and IV were 5 cases, 43 cases and 16 cases, respectively, as shown in Table 1. The distribution of age, operation time, and intraoperative blood loss is shown in Figure 2, which provides a visual representation of individual patient data.

**Table 1:** Clinicopathological features of patients with locally advanced gastric cancer.

Indicator	Cases
Gender	
Male	34
Woman	30
Age (yr)	57.28 ± 12.27
Operation mode	
Combined pancreatectomy	25
Combined splenectomy	20
Combined hepatectomy	12
Combined pancreaticoduodenectomy	5
Combined pancreaticoduodenectomy with right hemicolectomy	1
Combined colectomy	1
Operation time (min)	159.64 ± 25.19
Intraoperative blood loss (mL)	Median 150 (range: 30–1600)
TNM staging	
II staging	5
III staging	43
IV staging	16
Histopathology	
Adenocarcinoma	38
Adenosquamous carcinoma	7
Signet-ring cell carcinoma	13
Squamous carcinoma	3
Undifferentiated carcinoma	3

### 3.2. Follow-up results

**Figure 3.**
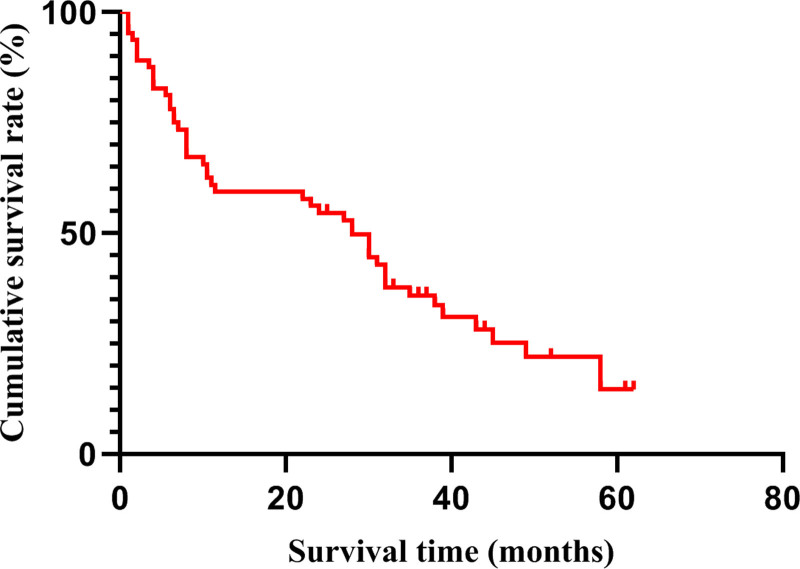
Postoperative survival curve of patients with locally advanced gastric cancer treated by EMR. EMR = extended multi-organ resection.

Short-term results: All 64 patients achieved R0 resection, and all patients were followed up for a median of 52 months, among which 47 patients died (73.44%) and 17 patients (26.56%) were still alive. Postoperative complications occurred in 18 cases (28.13%), including pancreatic fistula in 5 cases, anastomotic fistula in 5 cases, duodenal stump fistula in 3 cases, incision infection in 2 cases, abdominal infection in 1 case, subphrenic effusion in 1 case and liver and kidney failure in 1 case. The 30-day mortality rate was 4.69% (3/64), of which 1 case died on the 8th day due to postoperative abdominal infection, 1 case died because of anastomotic leakage and poor prognosis after exploratory laparotomy on the 13th day after operation, and both cases underwent gastric cancer combined with pancreaticoduodenectomy. One patient underwent combined subtotal hepatectomy, and died of liver and renal failure on the third day after operation due to massive bleeding during operation (Table 2). Long-term results: The average survival time of patients with locally advanced gastric cancer was 28.484 (95% CI; 22.965–34.004) months, and the median survival time was 28 months. The 1-, 3-, and 5-year survival rates were 59.4%, 35.9%, and 14.6%, respectively, as shown in Figure 3.

**Table 2:** Postoperative complications.

Local complications	Cases
Pancreatic fistula	5
Anastomotic fistula	5
Duodenal stump fistula	3
Incision infection	2
Abdominal infection	1
Subphrenic effusion	1
Liver and kidney failure	1
Total complication	18 (28.13%)
Clavien–Dindo category	
I	3
II	3
III	8
IV	1
V	3

## 4. Discussion

A considerable number of patients with gastric cancer have reached the advanced stage when they are diagnosed, and their adjacent organs have invaded. The factors that affect the prognosis of patients include the malignant degree of the tumor, the margin of the tumor and whether it has distant metastasis.^[9,10]^ For patients with limited metastasis and feasible surgical resection, patients with radical surgical resection can still obtain better prognosis. The prognosis of patients with distant metastasis outside abdominal cavity is generally poor, and the treatment is mainly neoadjuvant chemotherapy or combined molecular targeted therapy. Effective adjuvant chemotherapy, targeted therapy and immunotherapy can effectively reduce the tumor and reduce the unnecessary combined organ resection. However, some patients have surgical complications such as bleeding, perforation and obstruction during medical treatment, which makes surgery inevitable and even requires combined organ resection.^[11]^ All the cases included in this study were adjacent organ invasion, and 56% (36/64) patients were complicated with surgical complications such as digestive tract obstruction, bleeding and perforation.

Since Moynihan^[12]^ and Sugarbaker^[13]^ successively pointed out that overall resection can obtain a good long-term survival rate for locally advanced cancer, and the way of EMR for locally advanced cancer has gradually attracted people’s attention. EMR needs to completely remove the tumor and ensure that the tumor margin is negative, that is, the primary tumor focus, its invading organs and regional lymph nodes are cleared.^[14]^ Of course, EMR for patients with locally advanced gastric cancer also requires that the patient’s physical condition can tolerate major surgery, there is no distant metastasis (or metastasis is relatively limited and resectable), R0 resection is expected for tumor focus and involved tissues and organs, and sufficient effective lymph node dissection can be achieved.^[7]^ All the patients included in this study achieved R0 resection. Of course, the implementation of EMR for locally advanced gastric cancer not only maximizes the radical effect, but also has some problems, such as great trauma, many complications, slow postoperative recovery and high risk of death. In this study, 18 patients had postoperative complications, including 3 fatal complications. How to weigh the pros and cons, so that EMR can really benefit patients, not only need the surgeon to have rich clinical experience and superb surgical skills, but also need to build a multidisciplinary team including anesthesiology, ICU and oncology.

Pancreas and spleen are the most common organs invaded by advanced gastric cancer.^[15]^ In this study, pancreatic body and tail were invaded in 25 cases and spleen in 20 cases. We judge the combined splenectomy by evaluating whether the advanced gastric cancer directly invades or metastasizes to the spleen and its important blood vessels. The consensus at home and abroad points out that routine or preventive splenectomy is not needed when the spleen or the portal of the spleen is not involved, and the combined splenectomy aimed at thoroughly cleaning the lymph nodes at the portal of the spleen is not advocated. The splenectomy and method of preserving the spleen have been proved to be safe and feasible,^[16]^ and the D2 radical operation does not need routine splenectomy, and only when the lymph nodes at the portal of the spleen belong to the second station is it recommended to clean them. The second station lymph nodes of advanced proximal gastric cancer in the greater curvature of the stomach do not include the splenic hilar lymph nodes, but when the tumor invades the greater curvature of the stomach, the splenic hilar lymph nodes belong to the second station category.^[17]^ At first, the combination of advanced gastric cancer and pancreatectomy was also designed to facilitate the dissection of hilar lymph nodes. However, Brar et al^[18]^ found that the combination of pancreatectomy not only did not bring survival benefits, but increased postoperative complications. In this study, we performed combined tail pancreatectomy for patients with advanced gastric cancer whose tumor definitely invaded the pancreas or splenic artery trunk, and reserved the pancreas when the cancer tissue did not invade the pancreas but did not rule out lymph node metastasis around the splenic artery. Evidence of invasion is based on the results of intraoperative rapid freezing pathological examination to help judge. Locally advanced gastric cancer invading the liver is also common. In this study, 12 cases were combined with hepatectomy. When advanced gastric cancer directly invades the liver, combined hepatectomy should be actively considered to achieve the goal of R0 resection. However, for liver metastasis of gastric cancer, the indication of combined hepatectomy is still controversial. Some people think that when the liver metastasis is confined to 1 lobe, and there is no other organ metastasis or intrahepatic vascular invasion, combined resection of liver metastasis can benefit the survival of patients.^[19]^ It has also been recommended that combined hepatectomy is feasible for patients with single liver metastasis, with 1 or 2 liver lobes as the focus, and without vascular invasion and other organ metastasis.^[20]^ However, in clinical practice, liver metastasis of gastric cancer often presents multifocal or diffuse characteristics, and it is often combined with extensive metastasis of other organs, which makes it more difficult to choose treatment options. In this study, 1 case was given combined subtotal hepatectomy because of 3 liver metastases, but died of postoperative liver and renal failure due to massive bleeding during operation. Therefore, it is very important to fully evaluate PET/CT and MRI before operation, strictly grasp the indications of operation, and ensure that patients who plan to undergo combined hepatectomy have good liver reserve function. In this study, 5 patients with locally advanced gastric cancer were treated with combined pancreaticoduodenectomy because the cancer focus invaded the pancreatic head and duodenum (the initial part was more than 2 cm). When gastric cancer invades the duodenum or pancreatic head tissue, the scope is relatively limited and there is no distant metastasis, pancreaticoduodenectomy can be considered to achieve R0 resection. Combined pancreaticoduodenectomy for gastric cancer has better prognosis and higher survival rate than palliative surgery.^[21]^ However, this operation also has the disadvantages of large surgical trauma and many postoperative complications. In this study, 2 patients died of abdominal infection and anastomotic leakage after operation. In this study, another case of gastric cancer invaded the pancreatic head and the hepatic region of transverse colon, and underwent pancreaticoduodenectomy and right hemicolectomy during the operation, and anastomotic leakage occurred on the eighth day after operation. Considering that the patient had no obvious peritoneal irritation, he was given negative pressure irrigation and nutritional support and recovered. He was discharged smoothly on the 58th day after admission, and the patient was in good health after follow-up. There was also 1 patient whose stomach cancer invaded the transverse colon, causing obstruction, and was combined with transverse colon resection during operation. After follow-up, the prognosis of the patient was good. When advanced gastric cancer breaks through the large curvature and invades the transverse colon and its mesentery, the combined resection of transverse colon and its mesentery is not complicated and proved to improve the long-term survival.^[22]^

The indications of combined organ resection in our center are strictly as follows: Advanced gastric cancer or its lymph nodes invade the surrounding tissues, with relatively limited scope and no distant metastasis; There is distant metastasis, but the tumor types (such as poorly differentiated adenocarcinoma, mucinous adenocarcinoma, etc) are not sensitive to conservative medical treatment including radiotherapy and chemotherapy, so the prognosis of medical treatment is not ideal; Surgical complications, such as advanced gastric antrum cancer with pyloric obstruction and/or jaundice, colon bleeding and obstruction caused by gastric cancer invading colon, etc. Surgery combined with organ resection and tumor reduction can provide sufficient time window and treatment space for subsequent treatment. For example, resection of gastric cancer combined with partial hepatectomy can improve the spatial efficacy of subsequent hepatic artery intervention or radiofrequency ablation of liver. Pancreatoduodenectomy for advanced gastric antrum cancer with jaundice can relieve jaundice and provide sufficient time for later chemotherapy or immunotherapy. Patients can generally tolerate combined organ surgery and benefit from it.^[23]^

It is generally consistent with the previous research reports.^[24]^ Patients receiving EMR in this study have a high risk of complications, and the incidence of surgical complications is 28.13% (18/64), including 5 cases of pancreatic fistula, 5 cases of anastomotic leakage, 3 cases of duodenal stump fistula, 2 cases of incision infection, 1 case of abdominal infection, 1 case of subphrenic effusion, and 1 case of liver and kidney failure. Therefore, on the 1 hand, the principle of radical resection of tumor should be strictly followed during the operation, and the focus and organs should be removed in 1 piece, so as not to separate and cut off the tumor from the invaded organs, and at the same time, R0 resection with negative margin should be ensured; In addition, the complications of massive hemorrhage and organ tissue injury should be actively prevented and dealt with decisively during the operation, and the operator should carefully identify and dissect to avoid massive ligation and blind cutting of “unknown tissue”; Finally, we should actively build a multidisciplinary team covering gastrointestinal surgery, hepatobiliary and pancreatic surgery, urology, cardiothoracic surgery, gynecology, anesthesiology, etc, so as to make an accurate surgical plan before operation and successfully complete the combined organ resection operation through clear division of labor and skillful cooperation during operation.

Previous studies have reported that patients with locally advanced gastric cancer who undergo EMR have a higher survival rate than those who receive palliative surgery,^[6,25–27]^ and this study validated this conclusion with a 5-year survival rate of 14.6%. The median survival time of patients in this study was 28 months, and the 1-year, 3-year, and 5-year survival rates after operation were 59.4%, 35.9%, and 14.6%, respectively, which was consistent with the results of Ozer and Min.^[23,25]^ Our 5-year survival rate is lower than that in some published studies (where the 5-year survival rate was as high as 27.2%),^[24]^ which may be related to the higher proportion of patients with pN3 stage in this study. The proportion of pN3 patients in this study was as high as 67%, while the proportion of pN3 reported in other literatures was significantly lower, which is also one of the key factors contributing to the lower overall survival rate of patients who underwent EMR. There were 3 cases of perioperative death among the included subjects. Combined organ resection has the characteristics of large surgical trauma, many complications, slow postoperative recovery and high mortality. Strengthening perioperative management is very important for patients to successfully pass through the critical period after operation and recover quickly. Before operation, we should actively deal with basic diseases and reasonably control blood sugar and blood pressure; After operation, we should strengthen vital signs monitoring and organ function maintenance, attach importance to nutritional support and rapid rehabilitation, correctly master the management of drainage tube, double cannula, gastric tube and other pipes, and effectively prevent complications such as abdominal bleeding, infection, anastomotic fistula, pancreatic fistula, biliary fistula and intestinal fistula. In this study, the good long-term results are largely due to the standardized and good management of EMR during perioperative period.

This study has some limitations. First of all, this is a retrospective study of small sample size in a single center; Secondly, when we screened the subjects, we recruited patients who underwent gastrectomy combined with adjacent organ resection, but did not conduct a separate study on the cases that had to undergo combined organ resection because of surgical complications. Despite its limitations, this study provides new evidence that patients with EMR undergoing R0 resection can achieve acceptable postoperative morbidity, mortality, and improved survival rate. Combined organ resection plays an important role in it. For some patients, EMR may be the only way to get a radical cure. Palliative combined organ resection is sometimes the last resort to relieve or alleviate symptoms and improve the quality of life. In the future, the application of EMR in locally advanced gastric cancer still needs to accumulate more evidence-based medical evidence so that more patients can benefit from it.

## Acknowledgments

The authors would like to thank all colleagues for data collection from the Department of General Surgery, Qinghai Provincial People’s Hospital.

## Author contributions

**Conceptualization:** Shengwen Li.

**Data curation:** Yaming Guo, Wenjun Zhu, Xinyi Wang.

**Software:** Shengwen Li.

**Validation:** Song Li, Baozhang Chen.

**Writing – original draft:** Beibei He.

**Writing – review & editing:** Shile Wu.

## References

[1] Sung H Ferlay J Siegel RL. Global cancer statistics 2020: GLOBOCAN estimates of incidence and mortality worldwide for 36 cancers in 185 countries. CA Cancer J Clin. 2021;71:209–49. 33538338 10.3322/caac.21660

[2] GBD 2017 Stomach Cancer Collaborators. The global, regional, and national burden of stomach cancer in 195 countries, 1990–2017: a systematic analysis for the Global Burden of Disease study 2017. Lancet Gastroenterol Hepatol. 2020;5:42–54. 31648970 10.1016/S2468-1253(19)30328-0 PMC7033564

[3] Tan Z. Recent advances in the surgical treatment of advanced gastric cancer: a review. Med Sci Monit. 2019;25:3537–41. 31080234 10.12659/MSM.916475 PMC6528544

[4] Okumura Y Yamashita H Aikou S. Palliative distal gastrectomy offers no survival benefit over gastrojejunostomy for gastric cancer with outlet obstruction: retrospective analysis of an 11-year experience. World J Surg Oncol. 2014;12:364. 25432703 10.1186/1477-7819-12-364 PMC4364098

[5] Martin RC 2nd Jaques DP Brennan MF Karpeh M. Achieving RO resection for locally advanced gastric cancer: is it worth the risk of multiorgan resection? J Am Coll Surg. 2002;194:568–77. 12025834 10.1016/s1072-7515(02)01116-x

[6] Mita K Ito H Fukumoto M. Surgical outcomes and survival after extended multiorgan resection for T4 gastric cancer. Am J Surg. 2012;203:107–11. 21474116 10.1016/j.amjsurg.2010.12.007

[7] Xiao L Li M Xu F. Extended multi-organ resection for cT4 gastric carcinoma: a retrospective analysis. Pak J Med Sci. 2013;29:581–5. 24353581 10.12669/pjms.292.2898 PMC3809257

[8] Japanese Gastric Cancer Association. Japanese gastric cancer treatment guidelines 2010 (ver. 3). Gastric Cancer. 2011;14:113–23. 21573742 10.1007/s10120-011-0042-4

[9] Wang TB Zhou H Zhang XJ. Prognosis and related factors of patients with pathological complete response after neoadjuvant therapy for gastric cancer. Zhongguo Yi Xue Ke Xue Yuan Xue Bao. 2021;43:571–8. 34494528 10.3881/j.issn.1000-503X.13260

[10] Fugazzola P Ansaloni L Sartelli M. Advanced gastric cancer: the value of surgery. Acta Biomed. 2018;89:110–6. 30561428 10.23750/abm.v89i8-S.7897 PMC6502221

[11] Huang JY Xing YN Wang X. The prognosis value of lymphatic vessel invasion in pn0 gastric cancer patients with insufficient examined lymph nodes. J Gastrointest Surg. 2020;24:299–306. 30671803 10.1007/s11605-018-04101-z

[12] Birgisson H Olafsdottir EJ Sverrisdottir A Einarsson S Smaradottir A Tryggvadottir L. Screening for cancer of the colon and rectum. A review on incidence, mortality, cost and benefit. Laeknabladid. 2021;107:398–405. 34673541 10.17992/lbl.2021.09.65

[13] Sugarbaker ED. Coincident removal of additional structures in resections for carcinoma of the colon and rectum. Ann Surg. 1946;123:1036–46. 20987952

[14] Kirchberg J Weitz J. Intraabdominelle und retroperitoneale Sarkome [Intra-abdominal and retroperitoneal sarcomas]. Chirurg. 2016;87:255–66; quiz 267. 26920140 10.1007/s00104-016-0156-7

[15] Kitamura K Tani N Koike H. Combined resection of the involved organs in T4 gastric cancer. Hepatogastroenterology. 2000;47:1769–72. 11149053

[16] Guner A Hyung WJ. Advantages of splenic hilar lymph node dissection in proximal gastric cancer surgery. J Gastric Cancer. 2020;20:19–28. 32269841 10.5230/jgc.2020.20.e10 PMC7105411

[17] Huang C Jianxian L. Enlightenment and thinking of lymph node dissection in the splenic hilum of advanced proximal gastric cancer. Chin J Dig Surg. 2020;19:50–4.

[18] Brar SS Seevaratnam R Cardoso R Law C Helyer L Coburn N. A systematic review of spleen and pancreas preservation in extended lymphadenectomy for gastric cancer. Gastric Cancer. 2012;15(Suppl 1):S89–99. 21915699 10.1007/s10120-011-0087-4

[19] Jiankun H Linyong Z. Present situation and development of laparoscopic radical gastrectomy combined with organ resection. Chin J Gen Surg. 2017;11:457–60.

[20] Liu J Tong L. Principle and efficacy evaluation of laparoscopic radical gastrectomy combined with organ resection. Chin J Gen Surg. 2018;12:103–6.

[21] Xiaojian W Wenhua Z Ping L. Clinical analysis of combined organ resection in the treatment of advanced gastric cancer. Chin J Gen Surg. 2003;18:342–4.

[22] Maruyama K Kaminishi M Hayashi K. Gastric cancer treated in 1991 in Japan: data analysis of nationwide registry. Gastric Cancer. 2006;9:51–66. 16767357 10.1007/s10120-006-0370-y

[23] Min JS Jin SH Park S Kim SB Bang HY Lee JI. Prognosis of curatively resected pT4b gastric cancer with respect to invaded organ type. Ann Surg Oncol. 2012;19:494–501. 21837527 10.1245/s10434-011-1987-6

[24] Pacelli F Cusumano G Rosa F. Multivisceral resection for locally advanced gastric cancer: an Italian multicenter observational study. JAMA Surg. 2013;148:353–60. 23715879 10.1001/2013.jamasurg.309

[25] Ozer I Bostanci EB Orug T. Surgical outcomes and survival after multiorgan resection for locally advanced gastric cancer. Am J Surg. 2009;198:25–30. 18823618 10.1016/j.amjsurg.2008.06.031

[26] Kunisaki C Akiyama H Nomura M. Surgical outcomes in patients with T4 gastric carcinoma. J Am Coll Surg. 2006;202:223–30. 16427546 10.1016/j.jamcollsurg.2005.10.020

[27] Kim JH Jang YJ Park SS. Surgical outcomes and prognostic factors for T4 gastric cancers. Asian J Surg. 2009;32:198–204. 19892622 10.1016/S1015-9584(09)60395-X

